# Acylcarnitine esters profiling of serum and follicular fluid in patients undergoing *in vitro* fertilization

**DOI:** 10.1186/1477-7827-11-67

**Published:** 2013-07-17

**Authors:** Ákos Várnagy, Judit Bene, Endre Sulyok, Gábor L Kovács, József Bódis, Béla Melegh

**Affiliations:** 1 Department of Obstetrics and Gynecology, Faculty of Medicine, University of Pécs, Édesanyák u. 17, H-7624, Pécs, Hungary; 2 Department of Medical Genetics, Faculty of Medicine, University of Pécs, Szigeti u.12, H-7624, Pécs, Hungary; 3 Institute of Public Health and Health Promotion, Faculty of Health Sciences, University of Pécs, Vörösmarty u.4, H-7621, Pécs, Hungary; 4 Institute of Laboratory Medicine, Faculty of Medicine, University of Pécs, Ifjúság u.15, H-7624, Pécs, Hungary

**Keywords:** In vitro fertilization, Follicular fluid, Acylcarnitines, Oocyte number

## Abstract

**Background:**

L-carnitine-mediated beta-oxidation of fatty acids has a well established role in energy supply of oocytes and embryos. Disturbed carnitine metabolism may impair the reproductive potential in IVF and can serve as a biomarker of pregnancy outcome.

**Methods:**

Our study was performed between March 24, 2011 and May 9, 2011. We performed 44 unselected IVF cycles, (aged 23–40 years (mean: 32.3+/−5.1 years) and had BMI of 17.3-34.7 (mean: 23.80+/−4.9). Samples were also obtained from 18 healthy women of similar age admitted for minor elective surgery to serve as control for plasma carnitine profile. Serum and follicular fluid (FF) free carnitine (FC) and 20 major acylcarnitines (ACs) were measured by ESI/MS/MS method.

**Results:**

Serum FC and AC levels in IVF patients were comparable to those in healthy control women. In FF FC and short-chain AC concentrations were similar to those in maternal serum, however, the levels of medium-chain, and long-chain AC esters were markedly reduced (p<0.05). The serum to FF ratio of individual carnitine compounds increased progressively with increasing carbon chain length of AC esters (p<0.05). There was a marked reduction in total carnitine, FC and AC levels of serum and FF in patients with oocyte number of >9 and/or with embryo number of >6 as compared to the respective values of <9 and/or <6 (p<0.05).

**Conclusions:**

In IVF patients with better reproductive potential the carnitine/AC pathway appears to be upregulated that may result in excess carintine consumption and relative depletion of carnitine pool. Consequently, IVF patients may benefit from carnitine supplementation.

## Background

Normal oocyte maturation, fertility and embryo development is closely associated with energy metabolism [1-4]. The prominent role of fatty acids in energy supply to acquire developmental competence of oocyte and early embryos has been established [5-8]. However, growing body of evidences suggests, that non-esterified fatty acid supply in excess of its metabolic utilization results in fatty acid accumulation that may compromise oocyte maturation and developmental capacity of early embryo [9-11]. Interestingly, when free fatty acid composition of serum and/or follicular fluid (FF) was analyzed it became apparent that not isolated individual fatty acids, but rather physiologically relevant ratios and /or combinations of fatty acids cause significant dysregulation of cellular processes [8].

Free fatty acids are metabolised via beta-oxidation that is mediated by L-carnitine. L-carnitine is present in free and esterified forms in tissues and body fluids. It has multiple metabolic functions including transport of long-chain fatty acids into the mitochondria for beta-oxidation, transfer of short- and medium-chain acyl groups from the peroxisome to mitochondria, regulation of intracellular acyl-CoA/free CoA ratio and export of toxic acyl residues from the mitochondria [12-17]. Accumulation of acylcarnitines, (AC)s therefore is regarded as indicative of mitochondrial dysfunction and impaired cellular fatty acid metabolism [18]. The importance of L-carnitine in improving oocyte quality and reproductive performance has been demonstrated in animal and human studies [19-24].

On the basis of these observations it was intriguing to investigate further the relationship between carnitine profile and reproductive potential. In women who wants to conceive a child we can only analyze the serum carnitine profile and no FF samplaes are available, therefore patients receiving IVF can be an observational model. Also testing whether L-carnitine or individual carnitine esters alone or in combination can serve as potential biomarkers of pregnancy outcome.

The present study was conducted to determine the patterns of free carnitine (FC) and AC esters in serum and FF in women undergoing IVF. Attempts were also made to assess the properties of blood-follicular barrier by quantifying simultaneously the short-, medium- and long-chain ACs in the two distinct fluid compartments. In addition, composition of carnitine pools was related to indices of reproductive potential such as number of oocytes and that of viable embryos.

## Methods

### Ethical approval

The study was reviewed and approved by the Ethics Committee of the University of Pécs. Signed informed consent was obtained from all patients who participated in the study. The investigation conforms to the principles outlined in the Declaration of Helsinki.

### Patients

Our case–control study was performed between March 24, 2011 and May 9, 2011 in the Assisted Reproduction Unit, Department of Obstetrics and Gynecology, University of Pécs, Hungary. In this period we performed 44 unselected IVF cycles, in 42 cases we made transvaginal ultrasound guided aspiration of FF. In the remaining 2 cycles the stimulation was unsuccessful. The patients were aged 23–40 years (mean: 32.3±5.1 years) and had BMI of 17.3-34.7 (mean: 23.80±4.9).

The patients were recruited into this study according to the date of the procedure, so it was an unselected population. They presented with the following main infertility diagnosis: male factors (14, 33.3%), damaged or blocked Fallopian tubes (10, 23.8%), severe endometriosis (7, 16.7%) and unexplained infertility (11, 26.2%). These latter patients experienced six unsuccessful intrauterine inseminations previously.

Among the patients there were no diabetes mellitus (type I and II), or reduced glucose tolerance.

Superovulation treatment was started after the necessary examinations, such as cervical smear, serum hormone measurements (follicular stimulating and luteinizing hormones /FSH, LH/, prolactin, estradiol, progesterone, testosterone, thyroid-stimulating hormone) on the 3rd and 21st days of the unstimulated cycles, human immune-deficiency virus and hepatitis-B surface antigen screening, hysteroscopy and andrologic examination. Patient enrollment into IVF procedure was approved by two independent physicians.

### Control patients

During the study period samples were also obtained from 18 healthy women aged 25–40 years (mean: 33.4±5.2 years) and had BMI of 19.4-32.6 (mean: 25.01±4.7) admitted for minor elective surgery to serve as control for plasma carnitine profile.

### Controlled ovarian hyperstimulation

Inducing IVF GnRh agonist triptorelin (Gonapeptyl; Ferring®, Germany) was used in a long or short protocol, and the stimulation was performed with individual dosages of rFSH (Gonal-F; Serono® Aubonne, Switzerland), varying from 100 to 225 IU per day depending on the follicular maturation. The starting dose was adapted according to the BMI and the age. For patients with a previously known low response it was increased to a maximum dose of 300–350 IU daily. The follicular maturation was determined by ultrasound examination from the 6th day of the cycle, every other day. We changed the amount of the administered gonadotropins individually according to the size of the follicles. Ovulation was induced by injection of 250 μg of hCG (Ovitrelle; Serono®Aubonne, Switzerland) when at least two follicles exceeded 17 mm in diameter, and aspiration of FF was performed 36 hours later by ultrasonography-guided transvaginal puncture under routine intravenous sedation.

### Collection of follicular fluid

The oocyte collection was performed using Sonoace 6000C two dimensional real time ultrasound scanner equipped with 4–8 MHz endovaginal transducer. The oocyte collection was performed in G-MOPS™ medium (Vitrolife, Göteborg, Sweden).

FF from individual follicles was aspirated and after collecting the oocytes the fluid was centrifuged for 10 min at 1500 r.p.m. and the supernatants were frozen and stored at −70°C for future analysis.

### Fertilization methods

We performed the fertilization with intracytoplasmatic sperm injection (ICSI) depending on the andrological status (sperm count less than 15M/ml), the maternal age (> 35) and the number of the previous IVF cycles the patient had before (>2). The oocytes selected for ICSI were denuded with hyaluronidase and were assessed for maturity. Only metaphase II oocytes, identified by the presence of the first polar body, were chosen for fertilization. ICSI was performed 3–6 h after oocyte recovery in the medium G-MOPS™. The remained oocytes were fertilized with the conventional IVF method in a bicarbonate buffered medium (G-IVF™, Vitrolife®, Göteborg, Sweden). Fertilization was assessed 24 hours later in the medium G-1™ v5 (Vitrolife®, Göteborg, Sweden), the presence of two pronucleus signed the fertilization.

Embryo transfers were done 3–5 days after the oocyte retrieval. From day 3 to blastocyst stage we use the medium G-2™v5 (Vitrolife®, Göteborg, Sweden). According to the patient request we transferred one, two or three embryos. Cryopreservation of the remaining embryos was performed at this stage according to the Hungarian law. Progestogen supplementation was provided using 300 mg of progesterone 3 times a day (Utrogestan; Lab.Besins International S.A.®, Paris, France).

To evaluate the success of the treatment transvaginal ultrasound examination was performed 21 days after the embryo transfer to detect gestational sac.

### Measurements of FC and ACs

FC and all the ACs were determined by butyl-ester forms using isotope dilution mass spectrometry (MS) method in a Micromass Quattro Ultima (Manchester, UK) ESI triple-quadrupole mass spectrometer coupled with a Waters 2795 HPLC (Milford, MA, USA) system for sample introduction. For sample preparation 10 μl of serum or FF was used and a previously described procedure was followed [25]. During the ESI/MS/MS analysis FC and ACs were measured by positive precursor ion scan of m/z 85, with a scan range of m/z: 200?550. The applied capillary voltage, cone voltage and collision energy were 2.54 kV, 55 V and 26 eV, respectively.

Our mass spectrometry facility is a registered participant in the International Newborn Screening Quality Assurance Program organized by the Center for Disease Control and Prevention, USA [26].

Routine hormone measurements were performed by using commercially available immunoassay kits. FSH and LH were measured with the electrochemiluminescent assay of Roche Ltd. (Elecsys 2010), while beta-HCG was measured with radioimmunoassay (Laborexpert, Hungary).

### Statistical analysis

All statistical analyses were performed using SPSS, version 20 (SPSS Inc., Chicago, IL, USA). Normality of data was evaluated by Kolmogorov-Smirnov test. Variables are presented as median and interquartile range. Differences in carnitine ester concentrations between patient and control groups were analysed using the Kruskal-Wallis test. If changes were found to be significant, Mann–Whitney U test were performed. Data binning was applied in the case of oocyte and embryo number. This process is a data pre-processing technique used to reduce the effects of minor observation errors. Optimal binning thresholds, resulting into harmonic groups were offered by the statistical program. For correlation analysis between serum and follicular fluid values, the non-parametric Spearman’s bivariate test was used. A difference of p<0.05 was considered as significant.

## Results

### Carnitine profile measurements

**Figure 1:**
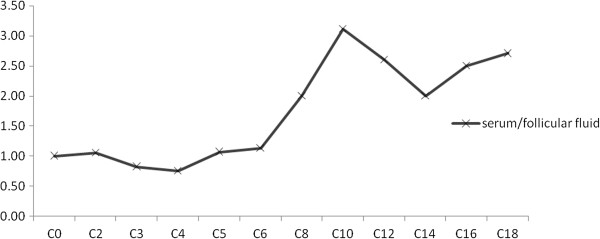
**Serum to follicular fluid ratio of individual acylcarnitines.** Serum to follicular fluid ratio of individual acylcarnitines as a function of the carbon chain length of acylesters.

**Figure 2:**
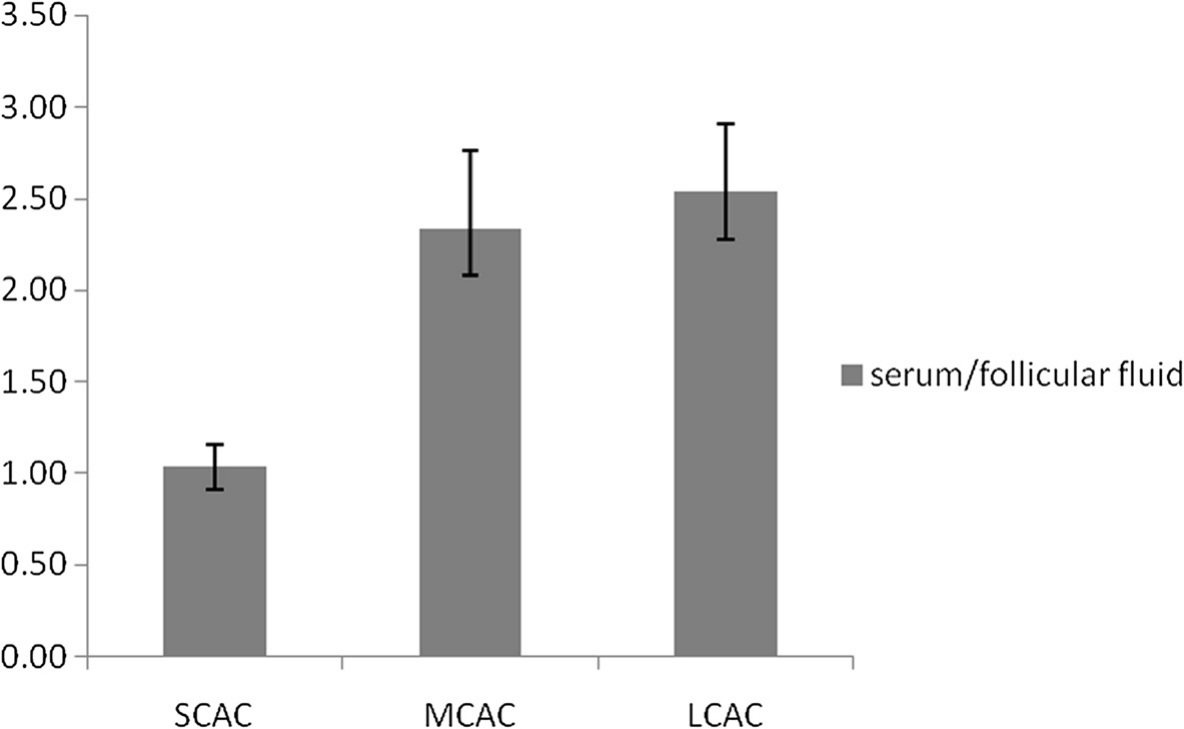
**Serum to follicular fluid ratio of the groups of acylcarnitines.** Serum to follicular fluid ratio of the groups of short-, medium-, and long-chain acylcarnitines as a function of the carbon chain length of acylcarnitines.

Serum and FF FC, individual and total ACs, as well as total carnitine concentrations of patients undergoing IVF are given in Table 1. For comparison the respective serum carnitine values for healthy control women was also presented. In the control group no follicular samples were available. As shown 20 distinct AC esters were detected in the serum and FF samples and there were no significant differences in serum carnitine levels between healthy subjects and IVF patients. However, analysis of FF carnitines revealed, that only the free and short-chain ACs were similar to those in maternal serum and the levels of medium-chain and long-chain AC ester levels were markedly lower. Accordingly, the serum to FF ratio of individual carnitine compounds increased progressively as the carbon chain length of carnitine esters increased (Figures 1 and 2). When FF carnitine profile was studied as a function of corresponding serum carnitines, a strong positive correlation was found between the two variables irrespective of their carbon chain length. Thus, serum free carnitine, total ACs, total carnitine, and total-, short-, medium- and long-chain ACs correlate highly significantly with the respective carnitine values in FF. Analysis of individual ACs, revealed that serum levels of C5-OH (short-chain), C12:1 (medium-chain) and C14, C14:1, C16 and C18 (long-chain) do not correlate with those in FF (Table 2). These observations suggest that FF carnitines mostly derive from plasma filtrate and AC esters with shorter carbon chain length more readily cross the blood/ovarian barrier than ACs with longer chain length.

**Table 1:** **Carnitine ester concentrations of serum and follicular fluids μmol**/**L** [**median** (**interquartile range**)]

**Analytes**	**IVF patients**		**Controls**
	**Serum**	**Follicular fluid**	**Serum**
	**Median (IQR) N=42**	**Median (IQR) N=42**	**Median (IQR) N=18**
free carnitine	28.056 (5.358)	27.880 (5.878)	27.416 (5.664)
Total acyl-carnitine	9.088 (1.727)	7.613 (1.887)	8.193 (1.413)
Total carnitine	36.309 (6.382)	35.895 (7.780)	36.298 (5.294)
AC/FC ratio	0.320 (0.079)	0.279 (0.049)	0.294 (0.071)
Total short-chain acylcarnitines	7.827 (1.505)	7.073 (1.760)	7.055 (1.500)
Total medium-chain acylcarnitine	0.688 (0.249)	0.307 (0.103) #	0.600 (0.228)
Total long-chain acylcarnitine	0.343 (0.069)	0.140 (0.033) #	0.338 (0.110)
**Short chain acylcarnitines**			
C2-carnitine	7.470 (1.300)	6.559 (1.740)	6.504 (1.514)
C3-carnitine	0.183 (0.073)*	0.204 (0.068) #	0.263 (0.090)
C4-carnitine	0.143 (0.058)*	0.160 (0.073)	0.180 (0.088)
C5-carnitine	0.053 (0.027)	0.052 (0.013)	0.050 (0.020)
C5-OH carnitine	0.025 (0.012)*	0.020 (0.007) #	0.033 (0.014)
**Medium chain acylcarnitines**			
C6-carnitine	0.027 (0.010)	0.020 (0.007) #	0.027 (0.007)
C8-carnitine	0.098 (0.043)	0.053 (0.024) #	0.097 (0.0459
C8:1-carnitine	0.037 (0.020)	0.030 (0.020)	0.038 (0.016)
C10-carnitine	0.215 (0.120)	0.070 (0.029) #	0.187 (0.087)
C10:1-carnitine	0.154 (0.061)	0.070 (0.030) #	0.150 (0.040)
C12-carnitine	0.057 (0.023)	0.020 (0.006) #	0.050 (0.023)
C12:1-carnitine	0.066 (0.021)	0.020 (0.010) #	0.063 (0.019)
**Long chain acylcarnitines**			
C14-carnitine	0.023 (0.007)	0.010 (0.003) #	0.022 (0.011)
C14:1-carnitine	0.053 (0.026)*	0.020 (0.007) #	0.045 (0.016)
C16-carnitine	0.087 (0.029)	0.040 (0.013) #	0.092 (0.039)
C18-carnitine	0.032 (0.013)	0.013 (0.007) #	0.037 (0.010)
C18:1-carnitine	0.092 (0.032)	0.033 (0.013) #	0.080 (0.032)
C18:2-carnitine	0.053 (0.020)	0.020 (0.007) #	0.055 (0.027)
**Dicarboxylic acylcarnitines**			
C4DC-carnitine	0.030 (0.013)	0.023 (0.010) #	0.028 (0.005)
C5DC-carnitine	0.086 (0.040)	0.052 (0.017) #	0.082 (0.025)

*In the control group no follicular samples were avaiable*.

^*^p<0.05 compared to control.

^#^ p<0.05 compared to control.

**Table 2:** **Correlation analysis between carnitine profiles of serum and follicular fluid** (**n**= **42**)

**Analytes**	**Correlation coefficient**	***p***
free carnitine	0.868	<0.001
Total acyl-carnitine	0.368	0.008
Total carnitine	0.775	<0.001
Total short-chain acylcarnitines	0.385	0.006
Total medium-chain acylcarnitine	0.487	0.001
Total long-chain acylcarnitine	0.366	0.009
**Short chain acylcarnitines**		
C2-carnitine	0.394	0.005
C3-carnitine	0.683	<0.001
C4-carnitine	0.529	<0.001
C5-carnitine	0.475	<0.001
C5-OH carnitine	0.209	0.092
**Medium chain acylcarnitines**		
C6-carnitine	0.433	0.002
C8-carnitine	0.607	<0.001
C8:1-carnitine	0.790	<0.001
C10-carnitine	0.590	<0.001
C10:1-carnitine	0.532	<0.001
C12-carnitine	0.225	0.076
C12:1-carnitine	0.218	0.083
**Long chain acylcarnitines**		
C14-carnitine	−0.041	0.398
C14:1-carnitine	0.185	0.120
C16-carnitine	0.240	0.063
C18-carnitine	−0.140	0.188
C18:1-carnitine	0.385	0.006
C18:2-carnitine	0.482	0.001
**Dicarboxylic acylcarnitines**		
C4DC-carnitine	0.251	0.055
C5DC-carnitine	0.388	0.006

### Carnitine profile and reproductive potential

The association of serum and FF carnitine status with the number oocytes in IVF patients is shown in Table 3. It can be seen that serum FC fraction decreased significantly (p<0.05), and there was a general tendency to decrease for each, the short-, the medium-, and long-chain ACs in patients with binned oocyte number in patients where 9 or more oocyte were collected. Of the long- chain ACs the decrease of C14:1 fraction also proved to be significant (p<0.05).

**Table 3:** **Carnitine profile according to the number of oocytes** [**μmol**/**L**] [**median** (**interquartile range**)]

	***Serum oocyte number***		***Follicular fluid oocyte number***	
	**≤ 9 N=29**	**>9 N=13**	**≤ 9 N=29**	**>9 N=13**
**Analytes**	**Median (IQR)**	**Median (IQR)**	**Median (IQR)**	**Median (IQR)**
free carnitine	28.667 (6.090)	24.897 (4.137)*	28.768 (4.408)	25.515 (4.307) #
Total acyl-carnitine	9.147 (2.268)	8.767 (0.960)	7.997 (1.825)	6.810 (1.908) #
Total carnitine	36.940 (6.965)	32.869 (4.616)*	37.098 (5.845)	31.622 (6.026) #
AC/FC ratio	0.316 (0.075)	0.345 (0.073)	0.280 (0.0499	0.278 (0.053)
Total short-chain acylcarnitines	7.860 (1.492)	7.787 (1.172)	7.373 (1.8459	6.343 (1.802) #
Total medium-chain acylcarnitine	0.715 (0.257)	0.648 (0.275)	0.313 (0.1209	0.277 (0.065)
Total long-chain acylcarnitine	0.347 (0.060)	0.312 (0.072)*	0.140 (0.027)	0.127 (0.040)
**Short chain acylcarnitines**				
C2-carnitine	7.393 (1.445)	7.470 (1.123)	6.833 (1.622)	5.880 (1.800) #
C3-carnitine	0.185 (0.075)	0.180 (0.063)	0.210 (0.070)	0.177 (0.053) #
C4-carnitine	0.143 (0.058)	0.140 (0.058)	0.170 (0.081)	0.153 (0.033)
C5-carnitine	0.058 (0.025)	0.050 (0.020)	0.053 (0.013)	0.050 (0.010)
C5-OH carnitine	0.023 (0.010)	0.030 (0.010)	0.020 (0.007)	0.020 (0.003)
**Medium chain acylcarnitines**				
C6-carnitine	0.027 (0.013)	0.027 (0.007)	0.020 (0.013)	0.020 (0.003)
C8-carnitine	0.100 (0.037)	0.093 (0.040)	0.060 (0.023)	0.053 (0.027)
C8:1-carnitine	0.035 (0.018)	0.040 (0.028)	0.030 (0.018)	0.030 (0.027)
C10-carnitine	0.215 (0.130)	0.215 (0.098)	0.070 (0.030)	0.055 (0.030) #
C10:1-carnitine	0.160 (0.063)	0.140 (0.033)	0.073 (0.030)	0.070 (0.023)
C12-carnitine	0.060 (0.023)	0.055 (0.018)	0.020 (0.007)	0.020 (0.003)
C12:1-carnitine	0.070 (0.023)	0.057 (0.020)	0.020 (0.012)	0.020 (0.010)
**Long chain acylcarnitines**				
C14-carnitine	0.023 (0.010)	0.020 (0.007)	0.010 (0.003)	0.010 (0.003)
C14:1-carnitine	0.057 (0.023)	0.040 (0.030)*	0.020 (0.010)	0.017 (0.007)
C16-carnitine	0.090 (0.027)	0.080 (0.037)	0.040 (0.007)	0.040 (0.013)
C18-carnitine	0.033 (0.010)	0.027 (0.013)	0.013 (0.007)	0.013 (0.010)
C18:1-carnitine	0.093 (0.032)	0.080 (0.037)	0.037 (0.010)	0.030 (0.010)
C18:2-carnitine	0.057 (0.013)	0.043 (0.020)	0.020 (0.007)	0.020 (0.013)
**Dicarboxylic acylcarnitines**				
C4DC-carnitine	0.030 (0.017)	0.030 (0.010)	0.023 (0.010)	0.027 (0.007) #
C5DC-carnitine	0.090 (0.037)	0.075 (0.023)	0.053 (0.022)	0.050 (0.010)

* p<0.05 compared to ≤ 9 oocyte number in serum.

# p<0.05 compared to ≤ 9 oocyte number in follicular fluid.

Similar pattern of distribution was observed for FF carnitine compounds. In patients with oocytes of 9 or more FC (p<0.05) and short-chain ACs (p<0.05) were significantly reduced, whereas the reduction of total medium-chain and total long-chain ACs did not reach statistical significance.

Table 4 compares serum and FF concentrations of FC and major ACs in IVF patients with binned embryo number of 6 or less embryo developed with those patients where more than 6 embryo developed. Higher embryo number is associated with significantly depressed FC (p<0.05) and total serum carnitines (p<0.05), albeit the decrease of ACs is insignificant (p<0.10), it appears to be consistent. These findings are mirrored in the carnitine profile of FF where in addition to the significant decrease of FC (P<0.05), ACs of various carbon chain length decreased moderately but unanimously in patients with embryo number of more than 6.

**Table 4:** **Carnitine profile according to the number of embryos** [**μmol**/**L**] [**median** (**interquartile range**)]

	***Serum embryo number***		***Follicular fluid embryo number***	
	**≤ 6 N=28**	**>6 N=14**	**≤ 6 N=28**	**>6 N=14**
**Analytes**	**Median (IQR)**	**Median (IQR)**	**Median (IQR)**	**Median (IQR)**
free carnitine	28.623 (6.217)	24.955 (4.592)*	28.698 (4.914)	25.887 (5.559) #
Total acyl-carnitine	9.148 (2.2399	8.605 (1.500)	7.905 (1.713)	6.938 (1.972)
Total carnitine	37.100 (6.500)	32.739 (4.708)*	36.912 (5.838)	32.956 (6.978)
AC/FC ratio	0.317 (0.0749	0.333 (0.072)	0.279 (0.047)	0.281 (0.065)
Total short-chain acylcarnitines	7.893 (1.4489	7.598 (1.676)	7.316 (1.633)	6.482 (1.900)
Total medium-chain acylcarnitine	0.722 (0.2669	0.619 (0.242)	0.318 (0.121)	0.276 (0.073)
Total long-chain acylcarnitine	0.346 (0.051)	0.313 (0.093)	0.140 (0.024)	0.127 (0.042)
**Short chain acylcarnitines**				
C2-carnitine	7.393 (1.445)	7.470 (1.123)	6.805 (1.525)	6.053 (1.844)
C3-carnitine	0.183 (0.083)	0.183 (0.058)	0.218 (0.071)	0.178 (0.048)
C4-carnitine	0.143 (0.063)	0.135 (0.052)	0.177 (0.085)	0.150 (0.032)
C5-carnitine	0.060 (0.023)	0.048 (0.019)	0.053 (0.013)	0.048 (0.012)
C5-OH carnitine	0.023 (0.010)	0.030 (0.010)	0.020 (0.007)	0.018 (0.003)
**Medium chain acylcarnitines**				
C6-carnitine	0.028 (0.013)	0.025 (0.008)	0.020 (0.013)	0.020 (0.003)
C8-carnitine	0.102 (0.036)	0.087 (0.046)	0.060 (0.023)	0.050 (0.025) #
C8:1-carnitine	0.037 (0.018)	0.035 (0.025)	0.030 (0.018)	0.030 (0.026)
C10-carnitine	0.220 (0.138)	0.206 (0.095)	0.073 (0.026)	0.055 (0.027) #
C10:1-carnitine	0.163 (0.067)	0.140 (0.030)	0.073 (0.028)	0.070 (0.020)
C12-carnitine	0.058 (0.022)	0.056 (0.020)	0.020 (0.004)	0.020 (0.006)
C12:1-carnitine	0.068 (0.025)	0.058 (0.019)	0.022 (0.013)	0.020 (0.010)
**Long chain acylcarnitines**				
C14-carnitine	0.023 (0.010)	0.022 (0.009)	0.010 (0.004)	0.010 (0.003)
C14:1-carnitine	0.056 (0.023)	0.042 (0.030)*	0.020 (0.011)	0.017 (0.007)
C16-carnitine	0.088 (0.026)	0.080 (0.0239	0.038 (0.008)	0.040 (0.012)
C18-carnitine	0.033 (0.010)	0.028 (0.016)	0.013 (0.007)	0.013 (0.009)
C18:1-carnitine	0.093 (0.029)	0.085 (0.040)	0.035 (0.011)	0.030 (0.013)
C18:2-carnitine	0.055 (0.015)	0.047 (0.020)	0.020 (0.007)	0.020 (0.012)
**Dicarboxylic acylcarnitines**				
C4DC-carnitine	0.028 (0.017)	0.030 (0.008)	0.023 (0.010)	0.027 (0.007) #
C5DC-carnitine	0.092 (0.040)	0.078 (0.024)	0.055 (0.021)	0.050 (0.013)

* p<0.05 compared to ≤ 6 embryo number in serum.

# p<0.05 compared to ≤ 6 embryo number in follicular fluid.

## Discussion

The present study provided evidences that serum carnitine profile of patients undergoing IVF is comparable to that of healthy women. FC and major AC esters can be detected in the FF, and the serum to FF ratio of individual carnitine compounds is inversely related to the carbon chain length of carnitine esters. Moreover, markers of reproductive potential (number of oocytes and embryos) appeared to be associated with reduction of FC and some individual ACs both in serum and FF.

In recent years considerable efforts have been made to identify potential biomarkers in FF to predict IVF outcome. FF serves as dynamic, physiological environment of maturing oocytes and embryos, therefore, it is assumed to reflect metabolic changes that occur during maturation. It has been shown to contain hormones, growth factors, reactive oxygen species, cytokines, apoptotic factors and several metabolic intermediates [27,28]. Targeted analysis of specific biomarkers or certain combination of biomarkers has revealed important correlation with oocyte quality and/or related embryo.

The recent introduction of metabolomic and proteomic profiling of FF demonstrated that combined use of a panel of biomarkers as opposed to a single biomarker proved to be a reliable estimate of pregnancy outcome [29-32].

Our present study was prompted by the observations that L-carnitine-mediated β-oxidation of fatty acids is an essential energy source for oocyte and embryo development [5-8]. In this regard Dunning et al. reported that inhibition of carnitine palmitoyl transferase I (CPT I), the enzyme that catalyzes the initial step of β-oxidation, with etomoxir impaired subsequent embryo development. On the other hand, upregulation of β-oxidation during oocyte maturation by L-carnitine increased oocyte developmental competence as manifested by the increased rate of cleavage to 2-cell stage [21]. It was also demonstrated by the same group that L-carnitine supplementation during in vitro 3D follicle culture significantly increased β-oxidation and markedly improved both fertilization rate and blastocyst development without altering survival, growth or differentiation of follicles [22]. The beneficial effect of L-carnitine on reproductive performance has been well- documented, it has been claimed, however, that in addition to its essential role in β-oxidation l-carnitine has the capacity to protect against oxidative stress, inflammation and apoptosis [20,23,24,33]. L-carnitine-related improvement of insulin resistance and glucose utilization may also contribute to the better IVF outcome [34]. However several animal studies investigated the impact of L-carnitine supplementation on oocyte quality and preimplantation embryo development [22-24,35-37], very limited data are accessible for the carnitine levels of human ovarian follicular samples [38,39] and no reports are available on their acylcarnitine profiles. In their study Montjean et al. could not observe correlations between the total carnitine content of the follicular fluid and either the circulating estradiol content of the serum or the outcome of the IVF attempt. Robker et al. investigated the L-carnitine concentrations in follicular fluid samples from women who were administered human chorionic gonadotropin. It is not known whether the L-carnitine level is regulated during the menstrual cycle by gonadotropin [38]. In our study in an attempt to further explore the relationship between IVF parameters and the composition of endogenous carnitine pool we determined the concentrations of FC and 20 major AC esters in serum and FF samples obtained from patients receiving IVF. Importantly, all individual ACs measured could be detected in FF, their concentrations, however, proved to be dependent on maternal serum concentrations and on the carbon chain length of acyl groups. These findings are consistent with the notion that FC and AC esters cross the blood-follicular barrier but the passage through this barrier is reduced as molecular weight and lipophilicity increases with the increasing carbon chain length. Moreover, serum and FF carnitine profiling revealed marked reduction in total carnitine, FC and AC levels in IVF patients with oocyte number of >9 and/or with embryo number of >6 as compared to those with the respective values of <9 and/or <6. Based on these results we suggest that the L-carnitine/AC pathway is upregulated and the actual carnitine pool is depleted in patients with better reproductive potential.

Mitochondrial transport of long-chain fatty acids for β-oxidation is achieved via the carnitine circle. This requires the consecutive action of CPT I, carnitine-AC translocase, and CPT II [40,41]. The coordinated increase in the activity of these enzymes may result in enhanced transfer of activated long-chain fatty acids across the mitochondrial membranes and in an increase of substrate availability for β-oxidation to meet the greater energy requirements of IVF patients with better developmental performance. It is conceivable that this accelerated process consumes excess carnitine and deprives carnitine pools.

While our study underlines the importance of carnitine-mediated β-oxidation in reproduction it is to be considered that women with disrupted carnitine cycle can conceive and proceed to successful pregnancy. Indeed, there are case reports of patients with adult-onset CPT II deficiency who become pregnant and went on to deliver preterm or full-term newborns [42-45]. In order to identify IVF patients at risk of functional L-carnitine deficiency further studies are to be conducted to measure simultaneously carnitine compounds and markers of free fatty acid metabolism.

## Conclusions

Carnitine profiling of women undergoing IVF provided suggestive evidences that L-carnitine metabolism is accelerated and the developmental competence of oocytes and early embryos can be optimalized by giving supplemental L-carnitine.

## Abbreviations

AC: Acylcarnitine; BMI: Body mass index; CPT: Carnitine palmitoyl transferase; FC: Free carnitine; FF: Follicular fluid; FSH: Follicular stimulating hormone; hCG: Human choriogonadotropin; ICSI: Intracytoplasmatic sperm injection; IVF: In vitro fertilization; LH: Luteinizing hormone.

## Competing interest

The authors declare, that they have no competing interest.

## Authors’ contributions

ÁV: Participated in designing the study, contributed to sample collection and critical discussion and drafted the manuscript. JB: Participated in designing the study, laboratory measurements and drafted the manuscript. ES: Conceived the study, participated in its design and critical discussion and drafted the manuscript. GLK: Contributed to data interpretation and statistical analysis. JB: Supervised the clinical part of the study, drafting the manuscript. BM: Supervised the laboratory part of the study, assisted with drafting the manuscript. All authors read and approved the final manuscript.
